# What we know about prostaglandin pathway dysfunction in chronic enteropathies: From endoscopy to molecular diagnosis

**DOI:** 10.1515/jtim-2026-0020

**Published:** 2026-03-26

**Authors:** Shuaizhi Ruan, Pengguang Yan, Qi Yan, Xiang Xu, Shuowen Zhang, Jing Wang, Ji Li, Jingnan Li

**Affiliations:** Department of Gastroenterology, Peking Union Medical College Hospital, Chinese Academy of Medical Sciences & Peking Union Medical College, Beijing, China; School of Life Sciences, Tsinghua University, Beijing, China; Department of Gastroenterology, Ruijin Hospital Affiliated to Shanghai Jiao Tong University School of Medicine, Shanghai Jiao Tong University, Shanghai, China

**Keywords:** nonspecific multiple ulcers of the small intestine, prostaglandin, NSAID-induced enteropathy, gene

## Abstract

Chronic enteropathies characterized by multiple superficial ulcers of the small intestine have long been under-recognized, particularly in their early stage. However, the occurrence of refractory occult bleeding and episodes of partial bowel obstruction in this disease severely impacts quality of life, while targeted therapeutic options remain limited. Although dysfunction of the prostaglandin metabolic pathway has been associated with mucosal damage, the underlying molecular mechanisms and potential therapeutic targets remain unclear. In recent years, in-depth investigations of nonsteroidal anti-inflammatory drug (NSAID)-induced enteropathy, along with the discovery of rare monogenic disorders affecting the prostaglandin metabolic pathway, have helped bridge this knowledge gap. A broader concept, termed “prostaglandin-associated enteropathy (PGAE)”, has since emerged, representing a monumental breakthrough in the differential diagnosis of nonspecific small intestinal ulcers. This narrative review focuses on prostaglandin metabolism and chronic intestinal ulcers, including NSAID-induced enteropathy and chronic enteropathies associated with solute carrier organic anion transporter family member 2A1 (*SLCO2A1*) and phospholipase A2 group IVA (*PLA2G4A*). By unraveling molecular connections and highlighting innovative therapeutic avenues, we aim to advance clinical management and improve the well-being and quality of life for patients with PGAE.

## Introduction

Traditionally, the small intestine (small bowel) distal to the duodenum has been regarded as the “black box” of the gastrointestinal (GI) tract because of its inaccessibility. Historically, small intestinal ulceration has been challenging to diagnose using radiologic imaging or even exploratory laparotomy. ^[1]^ Furthermore, small intestinal ulceration is a heterogenous entity with diverse etiologies, including Crohn’s disease, drug-induced enteropathies, congenital malformations (*e.g*., Meckel’s diverticulum), and rare causes, such as infections, Behcet’s disease, and ischemia.^[2, 3, 4]^ However, many small intestinal ulcers remain idiopathic.

Over the past few decades, the introduction of advanced diagnostic tools for the small intestine, particularly video capsule endoscopy (VCE)^[5]^ and double-balloon enteroscopy (DBE),^[6]^ has largely overcome the obstacles of noninvasively visualizing small intestinal lesions at close range. It is estimated that nearly 10% of healthy individuals have small intestinal ulcers,^[7]^ highlighting a potential public health concern. Therefore, understanding the molecular mechanisms underlying small intestinal ulcers is essential for improving therapeutic guidance.

One important cause of small intestinal ulcers is injury to the mucosal barrier. Protective factors for maintaining mucosal integrity include prostaglandins (PGs), particularly PGE_2_.^[8]^ PGE_2_ can promote mucus barrier formation, regulate mucosal epithelial turnover, and inhibit mucosal inflammation.^[9]^ However, current insights into PGE_2_-mediated mucosal protection primarily stem from upper GI findings, and the complex mucosal microenvironment of the small intestine makes its role in the mucosal barrier less well defined. With the re-evaluation of nonsteroidal anti-inflammatory drug (NSAID)-induced enteropathy—a relatively common but easily neglected condition—the critical roles of the small intestinal microbiota and bile acids in PG-associated small intestinal mucosal injury have emerged.^[10]^ Furthermore, burgeoning omics technologies have greatly extended our pathophysiological understanding of these ulcerative diseases at the molecular level. Mutations in genes involved in PGE_2_ synthesis and transport, such as *PLA2G4A*^[11]^ and *SLCO2A1*,^[12]^ have been identified. Changes in the PGE^2^ concentration gradient play a crucial role in intestinal mucosal repair,^[13]^ while pathologically high levels of PGE_2_ exhibit proinflammatory^[14]^ and profibrotic^[15]^ properties. Some researchers have suggested that NSAID-induced enteropathy, chronic enteropathy associated with *SLCO2A1* gene (CEAS), and cryptogenic multifocal ulcerous stenosing enteritis (CMUSE) are all linked to PG dysfunction, and have collectively termed them “prostaglandin-associated enteropathy” (PGAE).^[12]^ However, while CMUSE shares similar morphological characteristics with other enteropathies, only a subset of patients exhibit confirmed PG pathway dysfunction.^[16]^

In this review, we aim to elucidate the role of PGs in the development and repair of small intestinal mucosal lesions, with a particular focus on PGAE, offering new perspectives on the clinical diagnosis and treatment of these rare conditions.

## Biosynthesis and signaling of prostaglandins

**Figure 1:**
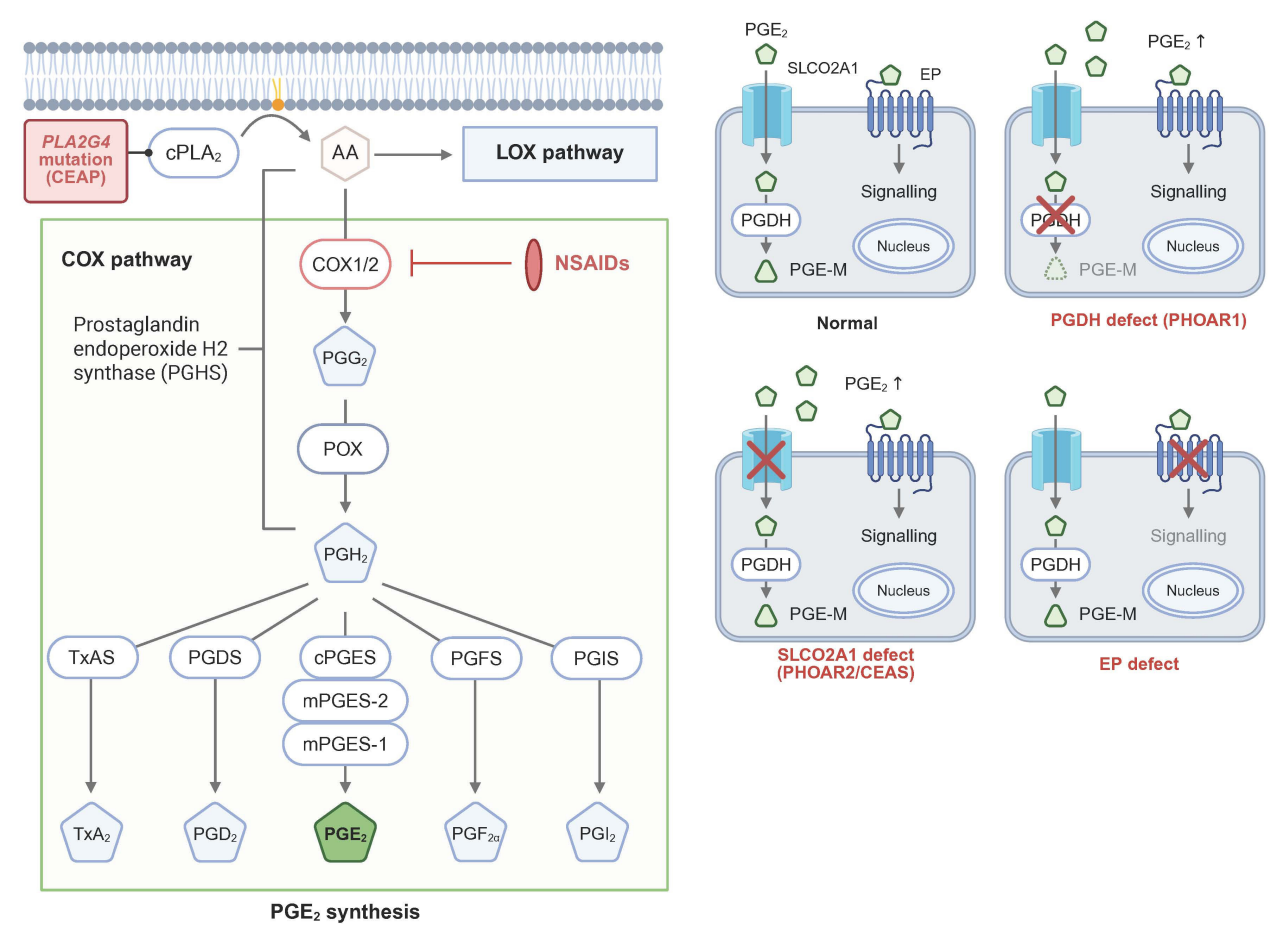
PGAE targets in the metabolic pathways of arachidonic acids. The biosynthesis of endogenous PGE2 begins with membrane phospholipids and proceeds sequentially via the cPLA2α, COX, POX, and PGE synthase enzymes. Notably, the gene encoding cPLA2α, PLA2G4A, is mutated in CEAP, and COX is the target site for NSAIDs. Once synthesized intracellularly, PGE2 is actively transported outside the cell, exerting its physiological effects by binding to the extracellular portion of EP receptors of the same or neighboring cells. Subsequently, PGE2 is reabsorbed into the cell via SLCO2A1 and degraded by 15-PGDH in the cytoplasm, thus terminating the signal transduction. Mutations in the genes encoding EP receptors, SLCO2A1, or 15-PGDH can lead to defective PGE2 signaling. cPLA2α: cytosolic phospholipase 2 alpha; COX: cyclooxygenase; CEAP: chronic enteropathy associated with the PLA2G4A; NSAIDs: nonsteroidal anti-inflammatory drugs; POX: peroxidase; SLCO2A1: solute carrier organic anion transporter family member 2A1; PGDH: 15-hydroxyprostaglandin dehydrogenase; PGE-M: PGE metabolites. Created in BioRender. Yan, Q. (2026) https://BioRender.com/cvoxrib.

Endogenous derivatives of arachidonic acids (AAs) are involved in various physiological and pathophysiological processes, including inflammation, carcinogenesis, and cardiovascular disorders.^[17]^ Metabolic pathways of AAs (Figure 1) commonly begin with membrane phospholipid hydrolysis by cytosolic phospholipase 2 alpha (cPLA2α, encoded by *PLA2G4A*) to produce AAs, which then diverge into two major pathways: cyclooxygenase (COX) and lipoxygenase (LOX). In the COX pathway, AAs are sequentially metabolized by COXs, peroxidase (POX), and either thromboxane synthase (to thromboxane A_2_ [TXA_2_]) or PG synthases (to PGs, including PGE_2_, prostacyclin [PGI_2_], PGD_2_, and PGF_2_α).^[18]^ Among these metabolites, PGE_2_ is the most well characterized in animal models, and is involved in all four classic signs of inflammation: redness, swelling, warmth, and pain.^[19]^ As early as the 1970s, gastroenterologists had identified PGE_2_ as a “cytoprotection” molecule that protects the gastric mucosa from harmful stimuli.^[8]^

Given the complexity of components involved in the metabolism and signaling of AAs, including enzymes, transporters, and receptors, the versatility of PGE_2_ extends far beyond these functions. Intracellular biosynthesis of PGE_2_ involves cPLA2α, COXs, and prostaglandin E synthases (PGESs). COXs have a constitutively expressed COX-1 isoform and an inflammation-induced COX-2 isoform, while PGESs have three: cytosolic (c)PGES, microsomal (m)PGES1, and mPGES2.^[18]^ Only mPGES1 is significantly induced by proinflammatory factors and functionally coupled with COX-2,^[20,21]^ in contrast to the constitutive expression of the other two isoforms.^[19,22]^ However, exceptions to this pattern have been observed. Alterations in PGE_2_ levels resulting from changes in one COX or PGES isoform may be offset by compensatory regulation of another isoform in certain cell and tissue types.^[23,24]^ This process, known as cross-regulation, suggests that these isoenzymes are not functionally isolated in pathophysiological processes. Newly synthesized PGE_2_ molecules, which are anionic at physiologic pH,^[25]^ must be actively transported to the extracellular space to function as autocrine or paracrine lipid mediators. ATP-dependent multidrug-resistance protein 4 (MRP4) is the key transporter mediating PGE_2_ efflux,^[25]^ although several studies have implicated exosomes or exocytosis of intracellular compartments, such as lysosomes,^[15]^ in this process. Subsequently, PGE^2^ molecules interact with four G-protein coupled receptor subtypes, namely EP1/2/3/4 (encoded by *PTGER1/2/3/4*, respectively),^[26]^ to trigger the intracellular signaling cascade. Because the distribution of these EP subtypes is tissue-specific, the roles of PGE_2_ molecules can vary considerably or even appear contradictory (*e.g*., proinflammatory or anti-inflammatory), depending on the context.^[18]^ It is important to clarify that the biological activity of PGE_2_, a classic inflammation-related factor, must remain transient to avoid homeostasis disruption and pathological inflammation. Researchers have established a two-step model for PGE_2_ metabolism (Figure 1) that involves solute carrier organic anion transporter family member 2A1 (SLCO2A1)-mediated PGE_2_ influx from extracellular fluid and oxidation to 15-keto PGE_2_ by cytoplasmic 15-hydroxyprostaglandin dehydrogenase (15-PGDH).^[27,28]^ This inactivates PGE_2_, leading to termination of the signaling cascade.

## PGE_2_ in small intestinal mucosal injury and repair

### Epithelial barrier defense mechanisms in the GI tract

**Figure 2:**
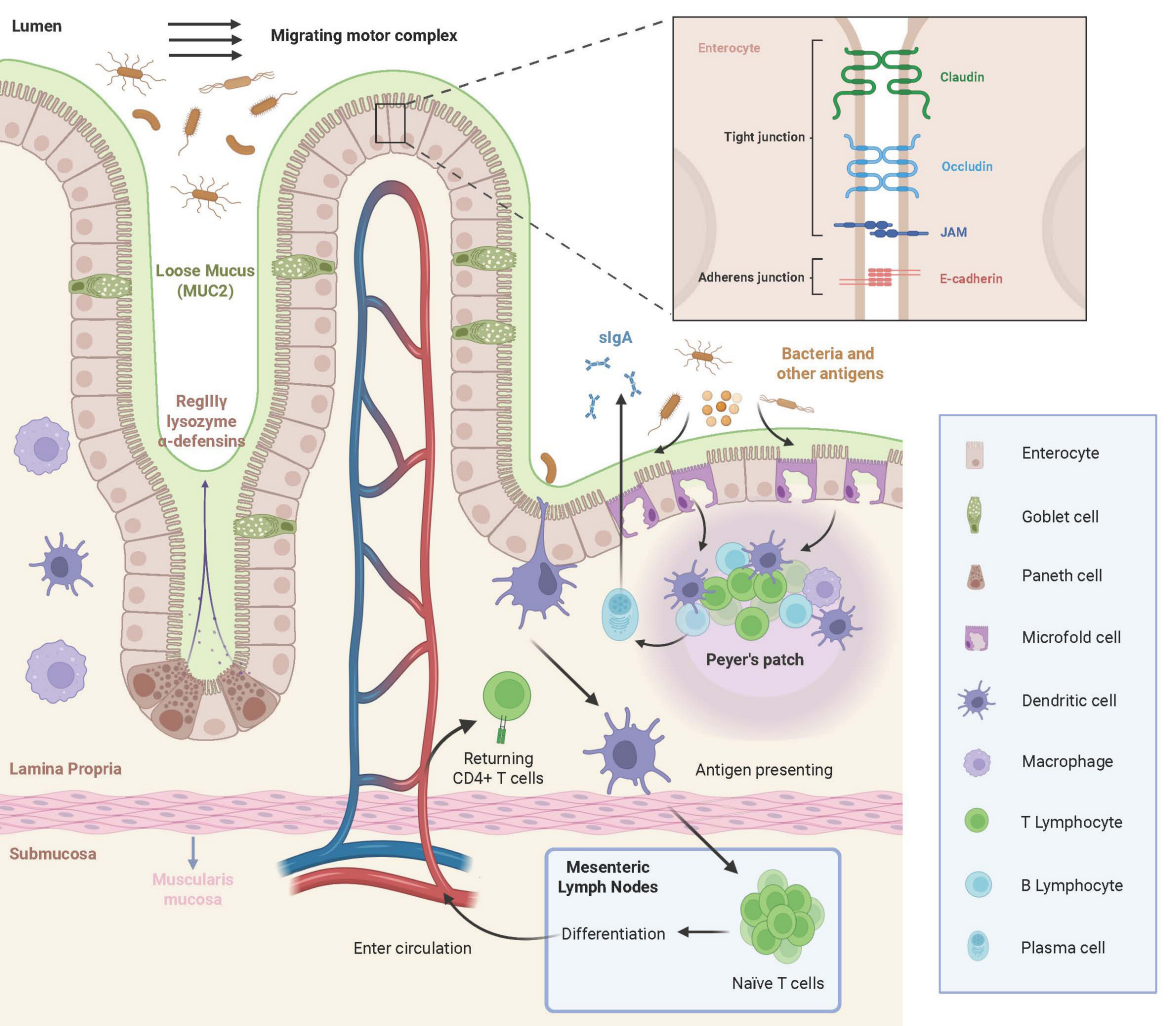
Mucosal defense system of the small intestine. The mucosal defense system of the small intestine comprises three compartments. The pre-epithelial compartment incorporates goblet cell-secreted MUC2-dominated mucus, reinforced by Paneth cell-derived antimicrobials (RegIIIγ, lysozyme, α-defensins) and intestinal migrating motor complexes. The epithelial compartment features tight junction- and adherens junction-sealed simple columnar epithelium. The post-epithelial compartment contains immune cells recruited from microvessels or inside Peyer’s patches, their secretory products, and extracellular matrix in the lamina propria. JAM: junctional adhesion molecule; sIgA: secreted immunoglobulin A. Created in BioRender. Yan, Q. (2026) https://BioRender.com/gkaukf9.

The epithelial lining of the GI tract displays significant regional variation in cellular composition, reflecting the unique physiological functions of each segment. Nevertheless, all segments of the GI epithelium serve a common function: acting as a barrier between the external environment and underlying tissues. In the small intestine, the selectively permeable epithelial barrier enables the absorption of large amounts of nutrients^[29]^ and facilitates interactions between commensal flora (despite their relatively low abundance) and immune cells to induce immune tolerance.^[30]^ It also plays a mucosal defense role, preventing undesirable substances and microorganisms from entering the circulation. This mucosal defense system has three components: pre-epithelial, epithelial, and post-epithelial^[31]^ (Figure 2). The pre-epithelial mucus is a continuous, bicarbonate-rich gel primarily composed of scaffolding mucins—mainly MUC2—secreted by goblet cells in the small intestine,^[32]^ along with smaller amounts of other glycoproteins and phospholipids, whose hydrophobic tails are arranged neatly to form the lumen-exposed interface. Unlike the colon, small intestinal mucus is relatively loose and detached from the epithelium, facilitating nutrient absorption.^[32]^ This layer is reinforced by the intense flushing action of the migrating motor complex^[32]^ and the release of antimicrobial peptides and enzymes from small intestine-specific Paneth cell granules.^[33]^ The epithelial component includes a simple columnar epithelial layer sealed by tight junctions and adherens junctions on the apical surface of the plasma membrane, where well-studied proteins such as occludin, claudins, and E-cadherin reside.^[30]^ The post-epithelial component is perhaps the most critical barrier for preserving deep mucosal integrity. This layer comprises abundant immune cells that migrate from microvessels or inside the Peyer’s patches within the lamina propria, along with their products (*e.g*., cytokines and immunoglobulins) and the extracellular matrix.^[31,33]^

## Roles of PGE2 in protection and repair of small intestinal mucosa

**Figure 3:**
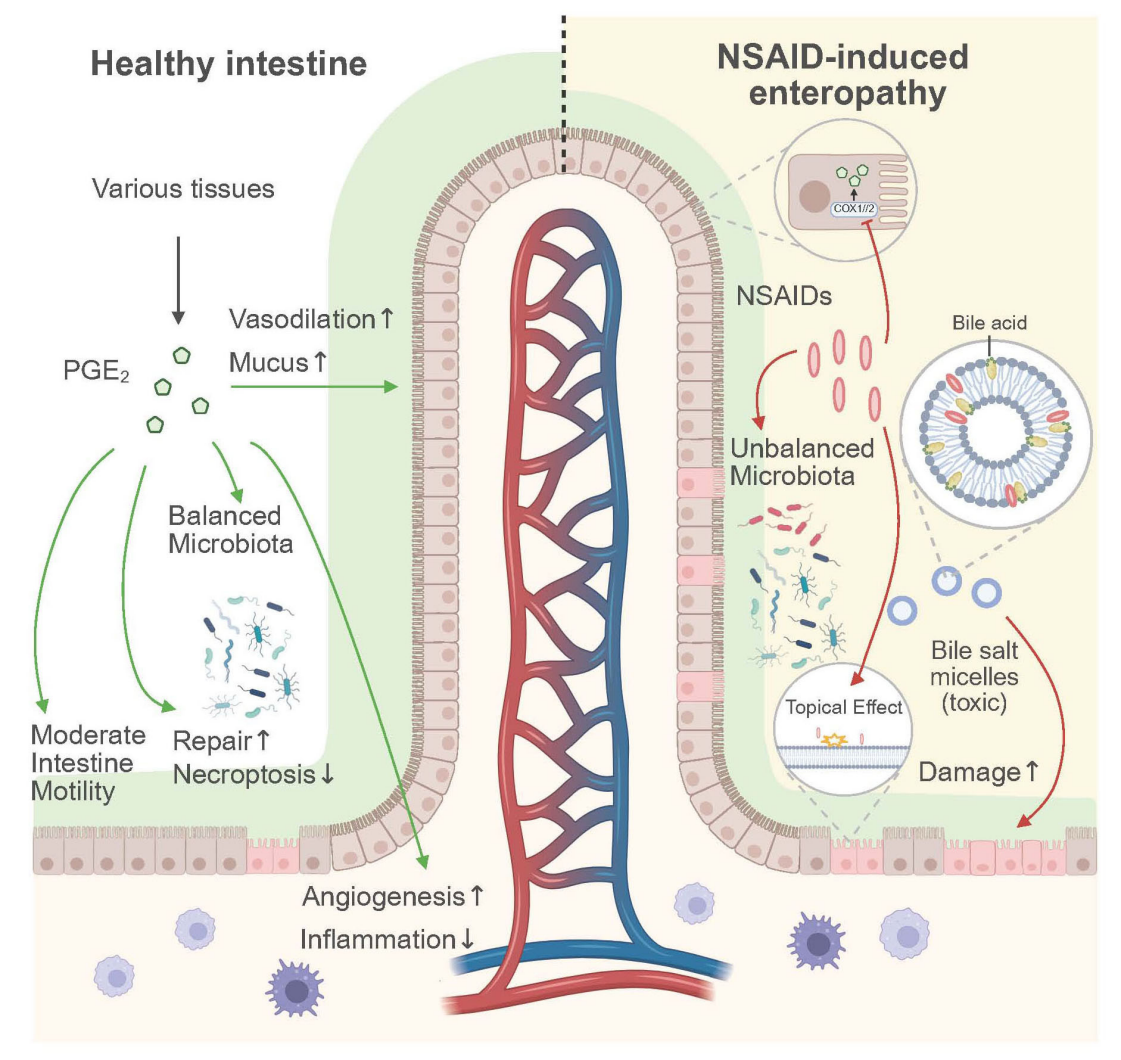
PGE2 roles in intestinal homeostasis and pathogenesis of NSAID-induced enteropathy. As a mucosal protective molecule, PGE2 moderates intestinal motility and dilates local microvessels to enhance mucus secretion, thereby alleviating mechanical wear on the mucosal epithelium. Upon mucosal injury, PGE2 mitigates inflammation by suppressing necroptosis of intestinal epithelial cells and chemotaxis of proinflammatory cells, while promoting mucosal repair through enhanced angiogenesis and concentration-dependent signaling modulation. In NSAID-induced enteropathy, COX inhibition leads to decreased endogenous PGE2 synthesis, thus diminishing the mucosal protective effects. The topical effects of NSAIDs, compounded by the formation of toxic bile salt micelles arising from their amphiphilic properties, further exacerbate mucosal damage. Intestinal luminal aggressors, including an unbalanced microbiota and bile salts, further predispose the mucosa to local inflammation. NSAIDs: nonsteroidal anti-inflammatory drugs. Created in BioRender. Yan, Q. (2026) https://BioRender.com/j20r363.

Acting as a mucosal protective agent, PGE_2_ is widely implicated in multiple processes related to small intestinal mucosal protection and repair (Figure 3). At the pre-epithelial level, PGE_2_ helps maintain moderate intestinal motility, thereby reducing mechanical wear on the mucus layer caused by excessive peristalsis.^[34]^ In rat experiments, COX inhibitor indomethacin and COX-1 selective inhibitor SC-560, but not COX-2 selective inhibitor rofecoxib, increased small intestinal contractions, which were counteracted by PGE_2_ or atropine.^[35]^ Additionally, PGE_2_ was found to increase mucus secretion by dilating local microvessels, an effect also observed with other vasodilatory agents (*e.g*., nitric oxide and hydrogen sulfide) in animal models.^[10_]_^ At the post-epithelial level, besides promoting vasodilation, PGE_2_ facilitates angiogenesis by upregulating vascular endothelial growth factor (VEGF) through activation of EP4 receptors on small intestinal epithelial cells,^[26]^ thereby regulating blood flow. Furthermore, PGE_2_ attenuates local inflammation through various immunomodulatory mechanisms in the lamina propria, such as reducing neutrophil chemotaxis,^[34]^ inhibiting CD4^+^ T-cell activation by enhancing the accumulation of polymorphonuclear myeloid-derived suppressor cells,^[36]^ and promoting M2 macrophage polarization.^[37]^ However, PGE_2_ derived from different cell types and secretion modes (*e.g*., autocrine or paracrine signaling) may exert varying effects on inflammation. For instance, T cell-derived autocrine PGE_2_ promotes colitis by reducing protective CD4^+^ RORγt^+^ cell responses, whereas PGE_2_ secreted by non-T lymphocytes in a paracrine manner inhibits colitis by promoting the expansion of FoxP3^+^ regulatory T cells.^[38]^

PGE_2_ exhibits diverse effects at the epithelial cell level. Early research revealed that the interaction of PGE_2_ with EP4 receptors upregulates the co-expression of chloride channel ClC-2 and tight junction proteins in intestinal epithelial cells (IECs),^[39]^ thereby strengthening the mucosal barrier *via* enhanced mucus secretion and intercellular junctions. Recent studies have further clarified the role of PGE_2_ in the lifecycle of IECs. In both human and mouse IECs, the interaction between PGE_2_ and EP4 receptors alleviates intestinal mucosal inflammation by inhibiting necroptosis mediated by receptor-interacting serine/threonine-protein kinase 3 (RIPK3) and mixed lineage kinase domain-like protein (MLKL).^[40]^ Upon mucosal injury, PGE_2_ plays a crucial role in various stages of epithelial repair. Epithelial restitution, the initial stage occurring within minutes to hours following injury,^[41]^ involves the rapid migration of specialized repair cells—termed wound-associated epithelial (WAE) cells^[42,43]^—from crypts surrounding the injured area to the injury site, covering the wound. During this phase, high concentrations of PGE_2_ synthesized by local mesenchymal cells act on EP4 receptors of intestinal stem cells (ISCs) and progenitor cells, leading to activation of the cAMP/protein kinase A (PKA) pathway and inhibition of glycogen synthase kinase 3β-mediated degradation of nuclear β-catenin. This process drives these cells to differentiate into WAE cells in a canonical Wnt-independent manner.^[42]^ Following transient epithelial restitution, WAE cells are replaced by mature columnar epithelium derived from the enhanced proliferation and differentiation of ISCs and progenitor cells over the subsequent hours to days, reconstructing structurally and functionally normal crypts.^[29,44]^ PGE_2_ can enhance the proliferative activity of ISCs, a process associated with upregulation of the Wnt-β-catenin signaling pathway,^[45]^ increased cyclin D1/2 expression in ISCs,^[46]^ and activation of the mitogen-activated protein kinase (MAPK)–C-X-C motif chemokine ligand 1 (CXCL1) pathway in intestinal macrophages.^[47]^ However, the differentiation process is inhibited by high concentrations of PGE_2_. Current evidence suggests that the secondary bile acid deoxycholate, a metabolite of the gut microbiota, downregulates cPLA2α activity and PGE_2_ synthesis in mesenchymal cells *via* the farnesoid X receptor, thereby permitting smooth stem cell differentiation and crypt reconstruction.^[13]^ Other studies suggest that, during the nonacute phase of injury, PGE_2_ induces ISCs to differentiate into goblet cells, promoting MUC2 secretion to reinforce mucosal barrier function.^[48]^

Given the complex role of PGE_2_ in maintaining intestinal mucosal homeostasis, factors that disrupt or exacerbate fluctuations in local or systemic PGE_2_ levels could contribute to small intestinal ulceration. Key factors of interest include medication usage, genetic defects, and abnormal content in the intestinal lumen. Next, we elaborate on these factors in the context of classic ulcerative diseases of the small intestine.

## NSAID-induced enteropathy: Extending beyond prostaglandins suppression

### NSAID-induced enteropathy: Underestimated risk and limited diagnosis

NSAIDs are among the most common over-the-counter and prescribed analgesics, with an estimated 30 million daily users worldwide.^[49]^ GI adverse reactions are perhaps the most well-known side effects of NSAIDs.^[50]^ Notably, during the COVID-19 pandemic, statistics indicated increased usage of NSAIDs and a rise in the incidence of upper GI bleeding.^[51]^ In pediatric patients presenting with upper GI bleeding, NSAID usage has reportedly surged nearly threefold.^[52]^ While these GI side effects are well known, the limited tools to assess the small intestine historically shifted attention toward NSAID-induced adverse effects in the upper GI tract (stomach and proximal duodenum), with less attention given to small intestinal damage. However, with the advent of new technologies such as VCE and DBE, multiple studies have confirmed that both short-term (2 weeks)^[53]^ and long-term (> 3 months)^[54]^ use of NSAIDs increases the incidence of small intestinal mucosal injury. Even low-dose aspirin (LDA; 81–325 mg per day),^[9]^ a commonly used NSAID for primary and secondary prevention of cardiovascular disease, has also been shown to cause small intestinal damage in both short-term^[55,56]^ and long-term^[57]^ users. Therefore, the potential risk of small intestinal lesions from long-term, high-dose NSAID use warrants close attention.

Despite the potential risk of NSAID-induced enteropathy, its diagnosis is challenged by the subtle onset of early symptoms, which may include occult or overt GI bleeding (anemia, melena, and hematochezia), hypoalbuminemia, and weight loss, preceding late-stage signs of intestinal stricture, such as abdominal pain, bloating, and recurrent vomiting.^[9,58,59]^ Severe cases may present with complications, including acute perforation, obstruction, or diverticulitis.^[60]^ However, these symptoms are nonspecific and lack sensitivity for detecting mild mucosal lesions in patients.^[9]^ Imaging techniques (*e.g*., computed tomography) often fail to definitively attribute findings such as intestinal wall thickening, mucosal hyperenhancement, or localized strictures^[61]^ to NSAID-induced enteropathy.

VCE and DBE have facilitated the early visualization of small lesions, establishing endoscopy as the diagnostic gold standard for NSAID-induced enteropathy when NSAID use is confirmed.^[58]^ Nevertheless, it is important to acknowledge that endoscopy is relatively expensive and invasive, with accessibility further limited by technological and equipment factors. Consequently, the search for simple, noninvasive biomarkers for small intestinal injury continues. Unfortunately, existing candidates, including fecal calprotectin,^[62]^ urinary chromium-51–labeled ethylenediaminetetraacetic acid (EDTA), and fecal indium-111–labeled neutrophils, have shown suboptimal performance in monitoring NSAID-induced enteropathy, thereby limiting their potential to replace endoscopy in diagnosing this condition.

Additionally, the endoscopic features of NSAID-induced enteropathy are diverse, with mucosal lesions appearing even after short-term drug use and persisting up to 18 months after discontinuation.^[64]^ Some scholars categorized these features as mucosal breaks (erosions or ulcers), villous atrophy, reddened folds, petechiae or red spots, and blood without an apparent source.^[53]^ Among these, mucosal breaks are the most common, accounting for approximately 40% of abnormal findings on VCE;^[53]^ however, the characteristics of these breaks are inconsistent, with ulcers presenting in various forms—circular, oval, longitudinal, and irregular.^[65]^ Based on these endoscopic morphological descriptions, researchers have established a five-score scale to assess the severity of NSAID-induced enteropathy,^[54]^ facilitating the development of individualized treatment strategies. The Lewis score is another widely utilized endoscopic grading method applicable to a range of inflammatory disorders of the small intestine,^[66]^ including NSAID-induced enteropathy and, more prominently, Crohn’s disease. NSAID-induced enteropathy typically involves multiple locations within the small intestine, with mucosal denudation more frequently occurring in the jejunum, ulcers in the ileum, and erosions scattered throughout the intestine.^[67]^ The duration of NSAID use may also influence endoscopic appearances. Chronic fibrosis of the small intestine resulting from long-term NSAID use may manifest as multiple short-segment circumferential strictures, known as diaphragm disease,^[61]^ a relatively specific feature of NSAID-induced enteropathy observed in only about 2% of patients.

### Impact of NSAIDs on prostaglandins pathways and intestinal homeostasis

NSAIDs exert their effects by inhibiting the activity of COX-1 and COX-2, thereby blocking the downstream synthesis of PGs. The consequent reduction in PGE_2_ can impair the small intestinal mucosal barrier (Figure 3). Given that COX-2 is typically induced during inflammation and that selective COX-2 inhibitors cause less gastroduodenal mucosal injury than nonselective COX inhibitors,^[68]^ their potential safety in the small intestine has also garnered interest. Current research indicates that short-term treatment with COX-2 inhibitors^[69]^ results in less small intestinal injury compared with nonselective NSAIDs, although this protective effect may diminish with longterm use.^[58,70,71]^

Apart from their enzyme inhibitory activity, all NSAIDs are amphiphilic organic weak acids,^[10]^ sharing substantial physicochemical similarities with phospholipid molecules. This similarity confers NSAIDs with surfactant-like properties, allowing them to disrupt the integrity of the intestinal mucus layer and epithelial cell membranes. This phenomenon, often referred to as the topical effect of NSAIDs,^[10]^ is further substantiated by the higher incidence of small intestinal mucosal damage observed with enteric-coated LDA compared with sustained-release LDA.^[72]^ Once the mucosal barrier is compromised and intestinal permeability increases, interactions between NSAIDs and luminal aggressors (mainly bile acids and gut microbiota) become pivotal in the pathogenesis (Figure 3). In rat models of small intestinal injury, early observations indicated that NSAIDs did not induce damage in bile duct-ligated rats.^[73,74]^ Additionally, certain NSAIDs (*e.g*., aspirin and nabumetone), which are not excreted *via* bile, do not cause injury when administered parenterally.^[75]^ However, coadministration of certain bile acids (*e.g*., glycocholate, taurocholate, and taurodeoxycholate) with indomethacin can potentially exacerbate damage.^[76]^ Similarly, germfree rats do not develop small intestinal injury following indomethacin injection,^[77]^ and the severity of injury is reduced in rats treated with broad-spectrum antibiotics (*e.g*., aztreonam^[78]^ and ampicillin^[79]^). Although the Toll-like receptor 4-mediated proapoptotic and proinflammatory effects of gut bacteria (particularly Gram-negative bacilli) on the intestinal epithelium have been previously reported,^[78]^ aforementioned findings suggest that this mechanism is not the sole contributor to mucosal damage in NSAID-induced enteropathy; bile acids also play a crucial role. The following mechanism for this process has been hypothesized:^[10]^ NSAIDs undergo conjugation at the carboxyl group in the liver to form acyl glucuronides, which are then excreted into bile *via* a specific hepatic canalicular transport pump. These conjugates are cleaved by bacterial β-glucuronidase in the intestine and partially reabsorbed in the distal ileum, re-entering the enterohepatic circulation. In animal models of NSAID-induced enteropathy with defective efflux pumps^[80]^ or β-glucuronidase inhibitor treatment,^[81]^ small intestinal mucosal damage is less severe than in untreated controls, providing robust support for this hypothesis. Repeated enterohepatic circulation prolongs the topical effects of NSAIDs, which may explain why more severe NSAID-induced enteropathy occurs more commonly in the mid and distal small intestine.^[67]^ Additionally, research has demonstrated that NSAIDs competitively bind to phosphatidylcholine in the presence of bile acids, resulting in the formation of more toxic bile salt micelles,^[82]^ which further compromise phospholipid integrity. Differences in luminal content between the stomach and small intestine also warrant consideration. Because bile acids and gut microbes, rather than gastric acid, are the primary luminal aggressors in the small intestine, proton pump inhibitors (PPIs) and potassium-competitive acid blockers—commonly used to treat NSAID-induced gastropathy—are largely ineffective against NSAID-induced enteropathy.^[9,59]^ In fact, they may even exacerbate enteropathy by reducing gastric acid secretion, which alters gut pH, leading to dysbiosis (particularly increases in Gram-negative bacteria and anaerobes^[59,83]^) or small intestinal bacterial overgrowth.^[84]^

### Clinical management strategies for NSAID-induced enteropathy

Although discontinuing NSAIDs is theoretically the most effective treatment for NSAID-induced enteropathy,^[85]^ this may interrupt management of the underlying disease and potentially worsen clinical outcomes.^[86]^ Therefore, coadministration of other drugs to prevent or treat NSAID-induced small intestinal mucosal injury is necessary (Table 1). Clinical studies have investigated several drugs that target two major therapeutic areas: enhancement of direct mucosal protection and modulation of luminal aggressive factors (primarily the gut microbiota). However, previous studies mainly focused on short-term, high-dose NSAID use in asymptomatic patients or healthy volunteers, with limited attention to the long-term prevention of small intestinal injury in chronic NSAID users.

**Table 1:** A Summary of therapeutics for PGAE

Disease	First-line therapy	Other therapies
NSAID-induced enteropathy	Discontinuation of NSAIDs with administration of mucosal protective agents (*e.g*., misoprostol, rebamipide)	Antibiotics, probiotics, anti-inflammatory agents (*e.g*., mesalazine, corticosteroids, immunomodulators, biologics), surgery
CEAS	Primarily supportive care (including blood transfusions, iron supplementation, and nutritional support), with potential efficacy of thalidomide/azathioprine in selected patients	Other anti-inflammatory agents (with limited efficacy), fecal microbiota transplantation, surgery
CEAP	Primarily supportive care (including blood transfusions, iron supplementation, and nutritional support)	Misoprostol, corticosteroids, Kangfuxin Liquid (a traditional Chinese medicinal formulation), surgery

Note: PGAE: prostaglandin-associated enteropathy; NSAID: non-steroidal anti-inflammatory drug; CEAS: chronic enteropathy associated with SLCO2A1 gene; CEAP: chronic enteropathy associated with PLA2G4A gene.

Mucosal protective agents are among the most established drugs for treating NSAID-induced enteropathy, acting through mechanisms such as enhancing local levels of mucosal PGs (or analogs), increasing mucosal blood flow, and restoring intercellular junctions.^[87]^ Misoprostol and rebamipide are prominent examples. Misoprostol, a synthetic PGE_1_ analog, is well known to reduce NSAID-induced GI damage.^[88,89]^ A recent multicenter, double-blind, randomized, placebo-controlled trial demonstrated its superiority in promoting small intestinal ulcer healing in patients taking LDA compared with placebo.^[90]^ However, misoprostol frequently induces diarrhea at therapeutic doses,^[59]^ limiting its use in NSAID-induced enteropathy. Rebamipide has shown comparable benefits in both treating^[91]^ and preventing^[87,92]^ NSAID-induced enteropathy with fewer side effects,^[93]^ making it more common in clinical practice. Other less commonly used agents include irsogladine, polaprezinc, teprenone (geranylgeranylacetone), egualen sodium hydrate, and ecabet sodium,^[58,94]^ all of which have demonstrated efficacy in clinical trials for preventing or treating NSAID-induced small intestinal injuries.

Therapies that modulate the gut microbiota primarily include antibiotics and probiotics. As crucial contributors to NSAID-induced enteropathy, Gram-negative bacteria and anaerobes are key targets for microbiome-based therapies. For example, metronidazole reduces small intestinal inflammation and bleeding in NSAID users,^[95]^ and rifaximin, a non-absorbed broad-spectrum antibiotic, significantly decreases the number and severity of small intestinal mucosal lesions observed on VCE.^[96]^ Gram-negative bacilli-targeted kanamycin and broad-spectrum antibiotic cocktails have also exhibited protective effects in animal models of NSAID-induced enteropathy.^[59]^ Probiotics have shown efficacy in multiple animal models,^[59]^ with recent clinical trials further substantiating these findings. In healthy subjects, compared with placebo, *Bifidobacterium breve* Bif195 significantly reduced VCE-detected small intestinal damage caused by acetylsalicylic acid,^[97]^ while a probiotic mixture comprising *Lactobacilli*, *Bifidobacteria*, and *Streptococcus salivarius* effectively decreased fecal calprotectin levels in indomethacin users.^[98]^ Among patients on chronic LDA therapy, the use of *Lactobacillus casei* significantly reduced small intestinal lesions detected on VCE,^[99]^ while the consumption of yogurt containing *Lactobacillus gasseri* demonstrated notable improvements in both clinical symptoms and endoscopic findings.^[100]^

A recent systematic review of NSAID-induced enteropathy incorporating 18 randomized controlled trials (RCTs) assessed the efficacy of multiple pharmacological agents, including rifaximin, probiotic formulations, the traditional Chinese medicine Muscovite, and several mucosal protective agents.^[94]^ The primary outcome of these trials was the change in the number of small intestinal lesions (pre- versus post-treatment) evaluated by VCE. Misoprostol was identified as the only significantly effective therapeutic agent for NSAID-associated small bowel injury, while rebamipide was found to be the only effective preventive agent. However, major limitations of these RCTs included small sample sizes, inadequate assessment of the clinical efficacy of probiotic formulations, and lack of long-term and real-world effectiveness evaluations. Therefore, further research on pharmacotherapies for NSAID-induced enteropathy remains warranted. Given the established efficacy of mucosal protective agents in NSAID-induced enteropathy, strategies targeting enhanced PGE_2_ bioavailability, such as 15-PGDH inhibitors,^[101]^ chemically modified PGE_2_ analogs with improved stability, and controlled-release exogenous PGE_2_ delivery systems,^[102]^ represent rational alternatives for future drug discovery.

Drugs used for other intestinal inflammatory disorders, such as inflammatory bowel disease and Behcet’s disease, have also been studied in NSAID-induced enteropathy. Sulfasalazine has been shown to reduce bleeding induced by NSAID intake;^[103,104]^ mesalazine granules reduce the severity of naproxen-induced small intestinal mucosal lesions on endoscopy;^[105]^ colchicine promotes healing of small intestinal mucosal injury in long-term NSAID users;^[106]^ and anti-tumor necrosis factor alpha (TNFα) agents alleviate small intestinal damage in rheumatoid arthritis patients treated with NSAIDs.^[107]^ Fecal microbiota transplantation (FMT), hydrogen sulfide- or nitric oxide-donor drugs,^[59]^ dopamine receptor agonists,^[108]^ dietary therapies,^[59]^ and herbal medicines^[109]^ have also been reported in animal models of NSAID-induced enteropathy.

## Small intestinal disorders associated with defects in PG metabolism-related genes

In the 1950s and 1960s, scientists from Spain, France, and Japan reported a series of cases of small intestinal disorders in rapid succession, based on findings from surgically resected bowel specimens.^[110, 111, 112]^ These cases were variously described as “primary nonspecific small-bowel ulceration”, “idiopathic ulcerations of the small intestine”, and “nonspecific small-bowel ulceration”.^[113]^ Despite these early reports, nonspecific small intestinal ulceration is relatively rare, with an estimated incidence of approximately 4 cases per 100,000 individuals.^[114]^

Among these nonspecific ulcerative conditions, two entities are clinically established: chronic nonspecific multiple ulcers of the small intestine (CNSU) and CMUSE. Both conditions present with persistent GI bleeding and protein loss.^[115]^ CNSU, which was first reported in six Japanese patients by Okabe and Sakimura in 1968,^[112]^ has never been diagnosed outside of Japan until 2019.^[116,117]^ While CMUSE, first described in cases reported by French researchers Rocha^[110]^ and Debray^[111]^ around 1960, has since been documented primarily in Europe, China, and South Korea.^[1]^ The diagnosis of these entities largely relies on imaging and endoscopic findings, and there is significant heterogeneity in treatment response, with female patients exhibiting steroid resistance having a poor prognosis. ^[118]^ The etiologies of CNSU and CMUSE remained unclear for decades, primarily because of limitations in research methodologies. These conditions have likely been frequently underdiagnosed or misdiagnosed, often mistaken for other ulcerative enteropathies.^[115,118]^

Recently, an increasing number of cases have shown familial clustering,^[1]^ accompanied by detection of gene mutations related to PG metabolism within these families. In 2014, Brooke *et al*. employed whole-genome single-nucleotide polymorphism (SNP) array analysis to identify a loss-of-function mutation in *PLA2G4A* gene, that led to abrogated production of AAs, the precursor of PGs, in two siblings severely affected by CMUSE.^[119]^ The following year, researchers identified a definitive genetic basis for CNSU involving *SLCO2A1* gene,^[12]^ which encodes a vital PG transporter, in several patients.^[120]^ They subsequently renamed CNSU as CEAS, and some scholars have suggested incorporating such genetic mutations into the diagnostic criteria for related enteropathies.^[115]^ Inspired by this naming convention, we propose that multiple nonspecific small intestinal ulcers with *PLA2G4A* mutations be classified as a new disease entity: chronic enteropathy associated with *PLA2G4A* (CEAP). Although this new category would include some cases previously classified under CMUSE or CNSU, it is not entirely synonymous with either, similar to CEAS. In the following sections, we will outline the characteristics of these conditions, including their pathogenesis, clinical features, endoscopic appearances, associated diseases, and treatments.

### Chronic enteropathy associated with the SLCO2A1 gene

*SLCO2A1*, the gene underlying the nomenclature of CEAS, is located on chromosomal 3q22.1-q22.2. It comprises 14 exons spanning ~ 95,000 base pairs and encodes the PG transporter responsible for PGE_2_ influx. Compared with CEAP, CEAS is more common and better understood, with recent reports providing reviews of documented CEAS cases.^[1,15,121]^ To date, 150 CEAS cases have been reported across 42 publications (Table 2), including 27 cases with consanguineous parentage and 129 cases involving a total of 45 confirmed *SLCO2A1* variants (Table 3). Demographically, the majority of these patients were female (male-to-female ratio of approximately 1:1.6) and from East Asia (Japan, China, and Korea). However, in the past two years, new cases have been reported from Turkey,^[122]^ France,^[123]^ Mexico,^[121]^ Algeria,^[124]^ India,^[125, 126, 127]^ and Sri Lanka,^[128]^ reflecting increasing awareness of this condition. CEAS typically presents with an insidious onset, most often manifesting as unexplained anemia or abdominal pain, and mainly occurs before the age of 40 (68/74 cases). The disease course is usually chronic and recurrent, with a diagnostic delay often exceeding 10 years. It is frequently misdiagnosed as small intestinal Crohn’s disease, CMUSE, Meckel’s diverticulum, *etc*. Laboratory findings frequently show anemia, positive occult blood in the stool, and hypoalbuminemia, but systemic inflammation markers (*e.g*., C-reactive protein and erythrocyte sedimentation rate) are typically not significantly elevated, as reported in CNSU. ^[113]^

**Table 2:** Clinical characteristics of CEAS cases (*n* = 150)

Characteristics	Item	Number of cases/Total documented cases (%)
Gender	Male	58/150 (38.67)
	Female	92/150 (61.33)
Age	CEAS symptom onset (years), median (Q1-Q3)	14.5 (7.5-24.0)
	CEAS diagnosis (years), median (Q1-Q3)	32.0 (17.5-44.5)
	Diagnosis delay (years), mean±SD	13.4 ± 10.8
Ethnicity	Japanese	83/150 (55.33)
	Chinese	29/150 (19.33)
	Korean	26/150 (17.33)
	Others	12/150 (8.00)
Genetics	Allele, homozygous	61/129 (47.29)
	Allele, heterozygous	4/129 (3.10)
	Allele, compound heterozygous	64/129 (49.61)
	Consanguinity	27/91 (29.67)
Chief complaints of CEAS	Anemia	119/126 (94.44)
	Hypoalbuminemia	92/126 (73.02)
	Abdominal pain	82/126 (65.08)
	Edema	31/126 (24.60)
	Diarrhea	27/126 (21.43)
	Weight loss or Developmental delay	22/126 (17.46)
	Melena or Hematochezia	21/126 (16.67)
	Ileus	17/126 (13.49)
	Intestinal perforation	4/126 (3.17)
Affected GI tract regions	Stomach	41/128 (32.03)
	Duodenum	60/128 (46.88)
	Jejunum	41/128 (32.03)
	Ileum excluding the terminal portion	115/128 (89.84)
	Terminal ileum	20/128 (15.63)
	Colon	13/128 (10.16)
Extra-Intestinal manifestations	PHO in male	27/58 (46.55)
	PHO in female	1/92 (1.09)
	Menstrual abnormality	7/92 (7.61)
	Myelofibrosis	3/150 (2.00)
Treatments	Surgery	80/150 (53.33)
	Mesalazine	32/150 (21.33)
	Corticosteroids	33/150 (22.00)
	Immunomodulators	35/150 (23.33)
	Anti-TNFα agents	21/150 (14.00)

CEAS: chronic enteropathy associated with SLCO2A1 gene; GI: gastrointestinal; PHO: primary hypertrophic osteoarthropathy; TNFα: tumor necrosis factor alpha.

**Table 3:** Variations of SLCO2A1 and PLA2G4A

Gene	DNA sequence	Protein change	Exon/intron	Variation type	ACMG Classification (based on ClinVar or gnomAD database)	Reference
*SLCO2A1*	c.97G>C	p.V33L	Exon2	Missense		[174,175]
	c.178G>A	p.E60K	Exon2	Missense	Pathogenic	[136]
	c.211G>C	p.G71R	Exon2	Missense		[152]
	c.253A>T	p.I85F	Exon3	Missense	Pathogenic	[124]
	c.289C>T	p.R97C	Exon3	Missense	Uncertain significance	[128]
	c.310G>A	p.G104R	Exon3	Missense	Likely pathogenic	[133]
	c.421G>T	p.E141X	Exon4	Nonsense	Pathogenic	[12,175]
	c.547G>A	p.G183R	Exon4	Missense	Pathogenic/Likely pathogenic	[12,121,136,174,175]
	c.621C>A	p.Y207X	Exon4	Nonsense	Pathogenic	[152]
	c.656C>T	p.P219L	Exon5	Missense	Uncertain significance	[122]
	c.664G>A	p.G222R	Exon5	Missense	Pathogenic	[12,135,174-177]
	c.724+1G>A	–	Intron5	Splicing		[116]
	c.770G>A	p.W257X	Exon6	Nonsense		[174,175]
	c.773G>A	p.W258X	Exon6	Nonsense		[125]
	c.830dupT	p.F277fs	Exon6	Frameshift	Pathogenic	[132,156,174,175]
	c.830delT	p.F277fs	Exon6	Frameshift	Pathogenic	[175]
	c.838C>T	p.R280X	Exon6	Nonsense		[133,157]
	c.855delA	p.A286fs	Exon6	Frameshift		[117,152,178]
	c.868_875dup	p.A293LfsX31	Exon7	Frameshift		[126]
	c.929A>G	p.D310G	Exon7	Missense		[152,178,179]
	c.940+1G>A	–	Intron7	Splicing	Pathogenic	[12,129,131,132,134, 135,156,174,175,176,177,180,181,182,183,184]
	c.941-1G>A	–	Intron7	Splicing		[133,136,151,152, 178]
	c.1106-1G>A	–	Intron8	Splicing	Pathogenic	[152]
	c.1106G>A	p.G369D	Exon9	Missense	Likely pathogenic	[133,152,153,178]
	c.1136G>A	p.G379E	Exon9	Missense		[152]
	c.1177delT	p.S393fs	Exon9	Frameshift		[152,178]
	c.1329_1344del	–	Exon10	Frameshift		[134]
	c.1350C>G	p.C450W	Exon10	Missense		[134]
	c.1372G>T	p.V458F	Exon10	Missense	Pathogenic	[12,175]
	c.1375T>C	p.C459R	Exon10	Missense		[152,178]
	c.1461G>C	p.L487L	Exon10	Synonymous		[135]
	c.1461+1G>C	–	Intron10	Splicing	Pathogenic	[12,174,175,177]
	c.1475G>A	p.C492Y	Exon11	Missense		[180]
	c.1559_1567dup	p.F520_I522dup	Exon11	Inframe insertion		[127]
	c.1622T>A	p.L541Q	Exon11	Missense		[133]
	c.1634delA	p.N545fs	Exon12	Frameshift	Pathogenic	[116]
	c.1649C>A	p.S550X	Exon12	Nonsense		[123]
	c.1660G>A	p.G554R	Exon12	Frameshift	Likely pathogenic	[123,152]
	c.1681C>T	p.R561C	Exon12	Missense		[136,152]
	c.1688T>C	p.L563P	Exon12	Missense		[181]
	c.1768delT	p.R590fs	Exon13	Frameshift	Pathogenic	[121]
	c.1771C>T	p.R591X	Exon13	Nonsense	Pathogenic	[125,152]
	c.1807C>T	p.R603X	Exon13	Nonsense	Pathogenic/Likely pathogenic	[12,117,126,129,131, 134,135,152,174,175, 177,178,185,186]
	c.1808G>C	p.R603P	Exon13	Missense	Uncertain significance	[130]
	c.1814+2T>G	–	Intron13	Splicing		[130]
*PLA2G4A*	c.331T>C	p.S111P	Exon5	Missense	Pathogenic	[11]
	c.1258G>C	p.E420Q	Exon12	Missense	Likely benign	[164]
	c.1454G>A	p.R485H	Exon14	Missense	Pathogenic	[11]
	c.1723G>C	p.D575H	Exon15	Missense	Pathogenic	[158]
	c.1952A>G	p.K651R	Exon16	Missense	Benign	[11]
	c.1952G>A	p.R651K	Exon16	Missense	Benign	[157]
	c.2118+4_2118 +7del	–	Intron17	Intron deletion	Pathogenic	[119]

–: Not available. ACMG: The American College of Medical Genetics and Genomics.

In CEAS, endoscopy typically reveals multiple shallow ulcers with well-demarcated borders—circular, longitudinal, oblique, geographic, or irregular in shape—often accompanied by multiple short-segment stenoses with intervening normal mucosa. The most frequently affected site is the ileum excluding the terminal portion (115/128), followed by the duodenum (60/128), stomach (41/128), jejunum (41/128), terminal ileum (20/128), and colon (13/128). Thus, initial endoscopic evaluation of CEAS heavily relies on VCE and DBE; however, there are frequent reports of capsule retention (9 cases in total) due to intestinal strictures.^[116,129,130,131,132,133,134]^ Histological analysis of biopsied or resected segments typically reveals ulcers confined to the mucosal or submucosal layers with a mixed infiltrate of nonspecific inflammatory cells, including plasma cells, lymphocytes, neutrophils, and eosinophils.^[122,133]^ Immunohistochemical staining for SLCO2A1 protein is generally negative, although not invariably.^[135,136]^ Radiologically, CEAS typically presents as multisegmented strictures or circumferential mural thickening and layered enhancement without involvement of the peripheral bowel structures.^[137]^ However, mild lesions may be challenging to discern on imaging. Overall, the clinical features of CEAS significantly overlap with those of NSAID-induced enteropathy and CMUSE/CEAP (see below), supporting our proposal to group these three conditions under the same disease category (Table 4).

**Table 4:** Clinical characteristics of CEAP cases (*n* = 7)

Patients	Gender	Age of CEAP onset (yr)	Age of CEAP diagnosis (vr)	Ethnicity	Familial clustering	*PLA2G4A* variation	*PLA2G4A* allele	Clinical manifestations	Affected Gl tract regions	Treatments	Reference
1	Male	0	45	American	No	c.331T>C c.1454 G>A c.1952A>G	Compound heterozygous	Anemia, Abdominal Pain, GIB, Intestinal Perforation	J, I	Surgery, Misoprostol	[11]
2	Male	4	48	Serbian	Yes	c.2118 + 4_2118 + 7del	Homozygous	Anemia, PU, GIB, Pyloric Stenosis, Hypoalbuminemia, Intestinal Volvulus and Multiple adhesions	S, D, J, I	Surgery, PPI, Corticosteroid	[119]
3	Female	2	45	Serbian	Yes	c.2118 + 4_2118 + 7del	Homozygous	Anemia, PU, GIB, Pyloric Stenosis, Intestinal Perforation, Intestinal Volvulus	S, I	Surgery, PPI	[119]
4	Male	2	27	Italian	Yes	c.1723G>C	Homozygous	Repeated Spontaneous Mucous Bleeding	S, D	PPI, Misoprostol	[158]
5	Female	2	27	Italian	Yes	c.1723G>C	Homozygous	Repeated Spontaneous Mucous Bleeding	S, D	PPI, Misoprostol	[158]
6	Male	31	33	Chinese	No	c.1952G>A	Homozygous	Anemia, Intestinal Perforation	J, I	Supportive treatments only	[157]
7	Male	14	20	Chinese	No	c.1258G>C	Heterozygous	Anemia, Abdominal Pain, Melena, Diarrhea, Intestinal Perforation, Ileus	I, c	Surgery, mesalazine, AZA, Kangfuxin Liquid	[164]

Regions: S-stomach, D-duodenum, J-Jejunum, l-ileum, C-colon; CEAP: chronic enteropathy associated with PLA2G4A gene AZA, azathioprine; Gl, gastrointestinal; GIB: gastrointestinal bleeding; PPI: proton pump inhibitor; PU: peptic ulceration.

Given the identified mutation sites, the fundamental pathophysiological change in CEAS involves loss of function of *SLCO2A1*, impairing the uptake of PGE_2_ for degradation^[138]^ and leading to an imbalance between intracellular and extracellular distribution of PGE_2_. The abnormal extracellular accumulation of PGE_2_ is reflected by elevated serum and urinary PGE_2_ levels.^[15,139]^ The proinflammatory and profibrotic effects of pathologically high local concentrations of PGE_2_ are probably central to CEAS pathogenesis. This pathogenic mechanism involves imbalances between pro- and anti-inflammatory processes,^[14]^ as well as between collagen synthesis and degradation,^[140]^ as demonstrated in animal models of pulmonary^[141]^ and cardiac fibrosis.^[142]^ However, it cannot fully explain why CEAS shares similar features with NSAID-induced enteropathy and CEAP, which are typically associated with reduced PGE_2_ levels. Considering the complex role of PGE_2_ in intestinal mucosal injury and repair, elevated PGE_2_ levels may have varied effects, depending on the specific microenvironment, cell type, or EP receptor subtype engaged.^[18]^ The role of PGE_2_ in organ and tissue fibrosis also remains controversial;^[140]^ notably, it reportedly exerts anti-fibrotic effects in murine models of chronic intestinal inflammation and fibrosis.^[143]^ Furthermore, the shared upstream substrates and similar chemical structures of various PG molecules suggest potential cross-reactivity among them. For example, besides PGE_2_, SLCO2A1 transporter substrates include other PG molecules with comparatively lower affinity, including PGD_2_ and PGF_2_α.^[138]^ Thus, the etiologies of these three diseases might involve cell-selective regulation or distinct downstream signaling pathways, warranting further investigation.

Any discussion of CEAS must address its association with primary hypertrophic osteoarthropathy (PHO), given the shared pathogenic mechanism involving impaired PGE_2_ degradation. The two-step PGE_2_ degradation process requires SLCO2A1-mediated transport and 15-PGDH–catalyzed oxidation, corresponding to the genetic variants in the PHO subtypes PHOAR2^[144]^ and PHOAR1,^[145]^ respectively (Figure 1), both of which involve disordered PG metabolism and excess PGE_2_ levels. Consequently, treatment with NSAIDs, particularly selective COX-2 inhibitors (*e.g*., etoricoxib),^[146,147]^ is effective for both subtypes. Elevated local levels of PGE_2_ in the skin, bone, and blood vessels contributed to PHO-related skin manifestations, including pachydermia (facial or scalp thickening), excessive sweating, acne, and seborrhea, which may result from epidermal hyperplasia and underdeveloped dermal tissue. In bone tissue, dysregulated formation and resorption can lead to periosteal new bone growth (periostosis) and delayed closure of cranial sutures. Prolonged PGE_2_ stimulation of blood vessels can also contribute to vascular abnormalities, including digital clubbing and patent ductus arteriosus.^[121]^ Among these, pachydermia, periostosis, and digital clubbing are considered the most common PHO triad.^[117,148]^ However, these two subtypes are not clinically identical. PHOAR1 patients are more likely to have severe skin involvement and distal phalanx resorption, while PHOAR2 patients, sharing the same causal gene as those with CEAS, often present with digestive tract bleeding.^[15]^ Demographically, PHOAR1 can present at birth or in early childhood,^[145]^ with no significant gender differences in symptoms. In contrast, PHOAR2 typically presents during adolescence (around age 15 years).^[139]^ Female PHOAR2 patients may present solely with CEAS, exhibiting minimal or no features of PHO;^[12]^ when present, PHO manifestations are more commonly observed in postmenopausal women.^[149]^ Previously, it was considered uncommon for male PHOAR2 patients to develop concurrent CEAS.^[12]^ However, our review of 58 male CEAS cases revealed that nearly half (27 individuals) were definitively diagnosed with PHO, suggesting that the relationship between these two conditions may be more intricate than previously thought, potentially representing two sides of the same coin. The unique demographic characteristics of PHOAR2 suggest that sex hormone levels^[15]^ or sex-related modifier genes^[150]^ may interact with PG signaling in the pathogenesis of PHOAR2/CEAS. Besides PHO, much less frequent extraintestinal manifestations of CEAS include menstrual abnormalities in women^[125,151,152,153]^ and myelofibrosis.^[116,117,118,119,120,121,122,123,124]^

Numerous clinical studies have demonstrated that, compared with healthy individuals, PHOAR1 patients have reduced urinary concentrations of PGE metabolites (PGE-M), whereas PHOAR2 patients exhibit significantly elevated PGE-M levels.^[15]^ This finding is perplexing, given that current theories predict reduced PGE-M levels in PHO patients across subtypes based on the shared impairment in PGE_2_ degradation. However, such findings imply the existence of additional PGE_2_ transporters or the possibility that SLCO2A1 mediates outward PGE_2_ transport. Recent studies have shown SLCO2A1 to be a crucial component of the Maxi-Cl channel,^[154]^ which is essential for intracellular ATP efflux and might also mediate the outward transport of anionic molecules, such as short-chain fatty acids and glutathione.^[155]^ The possibility that transcellular transport of PGE_2_ (anionic under neutral pH conditions) involves these molecules—all of which participate in intestinal mucosal homeostasis—is worth exploring.^[10,33]^ Elucidating the mechanisms underlying PGE_2_ transport may provide insight into the shared pathogenesis of CEAP, CEAS, and NSAID-induced enteropathy, all of which are characterized by multiple nonspecific small intestinal ulcers.

Because of its unclear pathogenesis, previous therapeutic strategies for CEAS have historically been adapted from Crohn’s disease management. However, pharmacotherapies for CEAS (Table 1), such as mesalazine, corticosteroids, immunomodulators, and biologics (including anti-TNFα agents), have shown limited efficacy overall. Among these, there are sporadic reports of improvement with azathioprine,^[122,130,134,156]^ while thalidomide has shown effectiveness, particularly in patients with severe bleeding.^[151, 152, 153]^ Anti-inflammatory therapies do not appear to be central to the management of CEAS patients, who are primarily affected by intestinal fibrotic stenosis and occult bleeding resulting from dilated small vessels beneath ulcerated areas. Most patients necessitate long-term symptomatic and supportive care, including blood transfusions, iron supplementation, and enteral nutritional support. In cases of severe bowel strictures, endoscopic balloon dilation is a viable option,^[152,153]^ although surgery remains the most effective short-term solution for symptom relief. Among the 150 cases reviewed, 80 patients underwent one or more surgical resections for affected intestinal segments. Novel therapies that have recently shown promise in managing the abdominal symptoms of CEAS include FMT^[133]^ and ruxolitinib,^[124]^ a selective JAK-1/2 inhibitor; however, the underlying mechanisms remain to be elucidated. Current CEAS management relies on empirical use of existing intestinal anti-inflammatory agents, but progress is limited by critical knowledge gaps in understanding the disease pathogenesis. Identification of therapeutic targets in *SLCO2A1*-deficient models could be pivotal for mechanism-based innovation.

### Chronic enteropathy associated with the PLA2G4A gene

Although *PLA2G4A* mutations have long been linked to small intestinal ulcers, to date, only seven confirmed patients can be classified as CEAP (Tables 3 and 4), and most documented cases of CMUSE/CNSU remain untested for this gene. In one case, the patient had mutations in both the *SLCO2A1* and *PLA2G4A* genes;^[157]^ however, the *PLA2G4A* “mutation” is actually a SNP with a relatively high frequency in the population, and structural biology analysis suggests that this variant does not affect cPLA2α function.^[11]^ Additionally, a case report of an Italian family^[158]^ identified a homozygous *PLA2G4A* missense mutation (c.1723G>C) in twins presenting with early-onset systematic mucosal bleeding (including epistaxis, gum bleeding, gastroduodenal bleeding, and hematuria) and postoperative hemorrhage. Although the twins did not undergo small-bowel endoscopy, their poor response to PPI treatment for gastroduodenal bleeding suggested that their bleeding might extend beyond the gastroduodenal region. Because *PLA2G4A* mutations are not currently considered the sole cause of CMUSE,^[16]^ the necessity of routine *PLA2G4A* genetic screening for all suspected CMUSE/CNSU patients remains uncertain. By contrast, CNSU patients are more commonly tested for *SLCO2A1* mutations.^[159]^

The limited number of documented CEAP cases makes it challenging to ascertain whether these patients represent the tip of the iceberg, or accurately reflect the broader clinical spectrum. However, as a subset of CMUSE/ CNSU, CEAP shares strikingly similar clinical features with these conditions, closely mimicking NSAID-induced enteropathy.

Notably, patients with CEAP seem to represent a more severe subset of CMUSE/CNSU, with most cases (5/7) presenting before age 5, unlike the typical onset in the fourth or fifth decade seen in CMUSE/CNSU.^[160,161]^ Furthermore, while these patients exhibited a chronic course, five also experienced acute complications, including intestinal perforation, obstruction, or torsion, to varying degrees. Four patients also displayed manifestations involving the stomach, duodenum, or even extraintestinal organs^[119,158]^—a rare occurrence in CMUSE.^[1]^ This suggests that patients previously diagnosed with CMUSE/CNSU may not constitute a homogeneous disease entity.

The *PLA2G4A* gene, located on chromosome 1q31.1 and spanning ~164,000 base pairs and 18 exons, encodes the 749-amino acid phospholipase cPLA2α.^[11]^ This enzyme acts farthest upstream in the PG metabolism pathway, providing essential substrates for synthesizing various inflammation-related molecules in the downstream COX and LOX pathways. Activation of cPLA2α is triggered by elevated intracellular Ca^2+^ levels or phosphorylation mediated by the MAPK or MNK1-related kinases.^[162]^ Mutations in *PLA2G4A* that result in loss of function lead to an absolute deficiency of PGE_2_ synthesis and consequent intestinal mucosal damage. Additionally, the synthesis of other protective, anti-inflammatory, and reparative molecules, such as PGD_2_, 15-deoxy-^△^12,14-PGJ_2_, and lipoxins,^[119]^ is inhibited, further exacerbating inflammation. Besides mucosal damage, *PLA2G4A* mutations may contribute to other key pathophysiological processes, including fibrosis,^[140]^ platelet inhibition,^[11]^ and abnormal neovascularization.^[163]^ Because different PGs exert distinct regulatory effects on fibrosis, an absolute reduction in their levels may disturb homeostatic balance, potentially leading to complications such as intestinal obstruction, adhesions, or volvulus.^[11,119]^ A reduction in platelet levels of thromboxane A_2_ (TXA_2_) impairs the physiological coagulation process, heightening the risk of systemic bleeding,^[158]^ including GI bleeding. Research on the tumor biology of *PLA2G4A* has revealed that hyperactivation of cPLA2α through phosphorylation promotes the migration and neovascularization of vascular endothelial cells while weakening their barrier function.^[163]^ Despite the deficiency of *PLA2G4A* expression in CEAP patients, this dysregulated signaling might still contribute to their characteristic small vessel dilation.

Treatment options for patients with CEAP are currently limited and largely empirical, primarily including supportive treatments, PPIs, corticosteroids, the PGE_1_ analog misoprostol, and surgical intervention (Table 1). Because small intestinal bleeding is not an acid-related disorder, PPIs have been ineffective in all documented cases.^[119,158]^ The only patient treated with corticosteroids showed no improvement.^[119]^ Misoprostol, though capable of providing some relief, does not prevent recurrent GI bleeding.^[11,158]^ Surgery is typically reserved for severe complications but is not curative, with many patients requiring repeated bowel resections.^[11,119,158]^ A recent study from China reported on a CEAP patient who was treated with Kangfuxin Liquid,^[164]^ a patented traditional Chinese medicinal formulation known for promoting wound healing and tissue repair. The patient exhibited symptom improvement following treatment; however, the long-term benefits remain uncertain and warrant further investigation.

Compared with CEAS and NSAID-induced enteropathy, CEAP targets an earlier step in the PG pathway, potentially complicating therapy because of broader systemic involvement. Considering the current scarcity of reported CEAP cases, future research should focus on defining its clinical and pathological features more comprehensively in additional patients, enabling the development of precision-based therapies.

### EP receptor-associated enteropathy

EP receptors are crucial membrane-bound proteins that bind PGs to initiate intracellular signaling pathways. Although loss-of-function mutations in these receptors may not directly impact the concentration or distribution of PGE_2_, as seen in PGAE, they undoubtedly disrupt its physiological functions. EP4 is the predominant EP receptor subtype expressed in the small intestine and colon.^[26]^ A recent study of ulcerative colitis patients undergoing ileal pouch-anal anastomosis surgery (*n* = 142) showed that SNPs in *PTGER4*, which encodes the EP4 receptor, are among the strongest predictors of Crohn’s disease-like complications, including fistulas, strictures, and granulomatous inflammation.^[165]^ This finding highlights the potential for EP4 receptor mutations to drive intestinal inflammation. Although no human cases of EP4 receptor (*PTGER4*) mutation-associated enteropathy have been reported, a *Ptger4* knockout mutation was strongly correlated with intestinal inflammation in mice. Histological analysis of the colonic mucosa in these knockout mice revealed reduced crypt depth, decreased secretory cells (goblet cells, enteroendocrine cells, and tuft cells), increased epithelial cell apoptosis, and enhanced infiltration of immune cells, including macrophages and CD4^+^ T cells in the lamina propria.^[166]^ At the molecular level, several proinflammatory cytokines and chemokines are upregulated.^[166,167]^ Furthermore, while these EP4-deficient mice exhibit increased susceptibility to dextran sulfate sodium (DSS)-induced colitis, administration of an EP4 agonist alleviates the disease severity.^[167]^ Beyond mucosal inflammation, animal studies using exogenous EP receptor agonists and antagonists have demonstrated that altered activation of EP receptors, particularly EP4, are closely associated with abnormal intestinal motility,^[168,169]^ secretory diarrhea,^[170,171]^ gut hormone secretion,^[172]^ and even the development of colorectal cancer.^[173]^

## Conclusion

Chronic enteropathies characterized by multiple superficial small intestinal ulcers and possible strictures comprise a heterogeneous group with poor prognosis and challenging diagnosis, owing to overlapping clinical and radiological features. However, advances in the understanding of the PG metabolic pathway have uncovered additional enteropathies potentially led to PG dysregulation. Given the critical role of PGs in maintaining epithelial barriers, precise regulation of PG metabolism is vital for intestinal mucosal homeostasis. Despite their heterogeneity, PG-related enteropathies share a common pathogenic mechanism: imbalance between mucosal injury and repair. Although more research is needed to elucidate the disease pathways, therapeutics targeting PG metabolism represent a promising strategy for restoring mucosal homeostasis.
